# Chlorophyll-a Pigment Measurement of Spirulina in Algal Growth Monitoring Using Portable Pulsed LED Fluorescence Lidar System

**DOI:** 10.3390/s22082940

**Published:** 2022-04-12

**Authors:** Jumar G. Cadondon, Prane Mariel B. Ong, Edgar A. Vallar, Tatsuo Shiina, Maria Cecilia D. Galvez

**Affiliations:** 1 Environment And RemoTe sensing researcH (EARTH) Laboratory, Physics Department, College of Science, De La Salle University Manila, 1004 Taft Avenue, Manila 0922, Philippines; prane.ong@dlsu.edu.ph (P.M.B.O.); edgar.vallar@dlsu.edu.ph (E.A.V.); maria.cecilia.galvez@dlsu.edu.ph (M.C.D.G.); 2 Division of Physical Sciences and Mathematics, College of Arts and Sciences, Miagao Campus, University of the Philippines Visayas, Miagao 5023, Philippines; 3 Graduate School of Engineering, Chiba University, Yayoi-cho, Chiba 263-8522, Japan; shiina@faculty.chiba-u.jp

**Keywords:** LED lidar, chlorophyll-a monitoring, fluorescence, remote sensing, applied optics

## Abstract

Chlorophyll-a measurement is important in algal growth and water quality monitoring in natural waters. A portable pulsed LED fluorescence lidar system based on the preliminary algal organic matter and pigments excitation–emission matrix (EEM) of commercialized AZTEC Spirulina powder at varying concentrations was developed. Fluorescence peaks from EEMs showed increasing intensity as the Spirulina concentration increases. Using this information, an LED fluorescence lidar with a wavelength of 385 nm, pulse width of 10 ns, and repetition frequency of 500 kHz was constructed for chlorophyll detection at 680 nm. Turbidity measurements were also conducted at 700 nm emission wavelength at the same excitation wavelength. Range-resolved fluorescence lidar signals from the portable pulsed LED fluorescence lidar system are highly correlated with the standard methods such as optical density at 680 nm (R^2^ = 0.87), EEM fluorescence chlorophyll-a pigment at 680 nm (R^2^ = 0.89), and corrected chlorophyll-a concentration (R^2^ =0.92). The F680/F700 lidar ratio was measured to provide a linear relationship of chlorophyll-a and turbidity in waters. The F680/F700 measurement showed strong correlations with Spirulina concentration (R^2^ = 0.94), absorbance at 680 nm (R^2^ = 0.84), EEM chlorophyll-a pigment at 680 nm (R^2^ = 0.83), and corrected chlorophyll-a concentration (R^2^ = 0.86). Results revealed that this new technique of chlorophyll-a measurement can be used as an alternative to other standard methods in algal growth monitoring.

## 1. Introduction

Algae are a diverse group of aquatic organisms that can conduct photosynthesis and are classified according to colors or pigments. Spirulina (*Arthrospira platensis*) is a blue-green algal culture, measuring 0.5 mm in length with a rod- or disk-like shape [1,2]. Algae habitats are dependent on nutrient-rich environments such as those with nitrates and mineral-rich alkaline. Effluents from sewage, residential housing, and industry can contribute to the degradation of water quality in coastal areas by adding an imbalanced amount of nutrients and sediment. Climate change and human activities have shown significant effects on algal biodiversity and surface water quality in natural waters [3]. Several water quality parameters are monitored regularly in the Philippines [4,5] to characterize river and lake waters—physicochemically, biologically, and bacteriologically—by which its acceptability is evaluated. There have been reports of poor water quality issues which are linked to algal blooms. Seasonal algal blooms frequently appear in the summer and have been observed among some beaches, river estuaries, and lakes in the Philippines [6].

The increased number of algal blooms in the surface region of the coastal waters greatly affects water quality and may increase sea surface temperature [7]. With the growing problems of harmful algal blooms (HABs) to human health and fisheries, methods for qualitative and quantitative analyses in natural waters are needed. Information on water quality assessments provided by standard methods is accurate; however, this may take a longer period to analyze. If changes in the water quality can be obtained before the water arrives in irrigation and reservoirs, people can apply preventative measures to possible illnesses that can be derived from these abnormal blooms [8,9,10].

Currently available methods for determining the microalgal population and pigments in waters are mostly post hoc analyses. There are direct and indirect measurements of chlorophyll-a pigment concentrations in waters. Direct measurements use dry weight analyses, which collects water samples and algal biomass is estimated [11]. On the other hand, indirect measurements using optical properties of water samples are used such as spectroscopy [12,13,14,15,16,17,18], flow cytometry [19,20,21], and high-pressure liquid chromatography [22,23]. In-situ fluorescence probes can provide an early warning of bloom development through fluorescence pigments at different emission wavelengths [18,24]. However, in-situ fluorescence probes can only measure at a fixed point.

Remote sensing systems obtain information about the object or areas at the Earth’s surface without direct contact with the object or area [25]. These systems use sensors with various sources of energy or radiation—namely, passive and active sensors. Passive sensors are remote sensing systems which measure energy that is naturally available [26]. Ocean remote sensing uses satellite data, which involves estimation of the concentration of suspended sediments, dissolved organic matter, and chlorophyll-a present in the water by measuring spectral quality variations [27]. Assessment of chlorophyll-a using unattended flow-through systems using seaborne [28,29,30], airborne [31,32], and spaceborne remote sensing [33,34] have been recommended to provide reliable data concerning the occurrence of algal blooms in coastal waters. However, algorithms developed from MODIS or SeaWIFS can also overestimate chlorophyll concentrations by at least 150% in the Baltic Sea, even in nonbloom conditions [35]. Cloud conditions also affect satellite data collection which leads to inconsistent water quality monitoring [36,37].

Active sensors are devices that require an external source of radiation to operate, unlike with passive sensors. These sensors simply detect and respond based on the input from the environment. Lidar is an active remote sensing technique which uses electromagnetic radiation in the optical range to detect a target, determine the distance of the target and the lidar, and deduce physical properties from optical parameters of the target based on the interaction of the radiation with the target through a phenomenon [38]. The design of the transmitting and receiving systems of the lidar is selected based on the physical phenomena being studied—such as scattering, absorption, reflection, and fluorescence. A fluorescence lidar system is a non-invasive remote sensing technique used for monitoring species in the atmosphere based on the detection of the fluorescence of atoms and molecules excited with a resonantly tuned laser [39]. With the aid of the fluorescence lidar technique, we can investigate the distribution and pattern of water components in natural waters. The first reported fluorescence lidar system was developed to map out the extent of oil–water interactions, chlorophylls, and tracer dye for hydrologic uses using an aircraft for environmental sensing [40]. Chlorophyll-a monitoring used fluorosensors [41], in-situ sensors [42], and laser-induced fluorescence systems [43,44,45]. Saito et al. (2014) started to develop a laser-induced fluorescence spectrum lidar system that monitors blue-green algae. A different study by Palmer et al. (2013) developed an ultraviolet fluorescence lidar system that simultaneously detect multiple water quality parameters in turbid conditions [46]. These transmitting systems used compact lasers with average pulse power of 15–20 W. With its high-power output, these emission intensities are not eye-safe to human operators.

Research using affordable remote sensing devices is one of the solutions for the problems in environmental monitoring. It also provides solutions on the sustainable development goals of the United Nations—specifically on clean water and sanitation and climate action [47]. Given the economic status of the country and its increasing environmental concerns, this study aims to present a unique measurement of chlorophyll-a concentrations in algal growth monitoring. The researchers developed a pulsed LED fluorescence lidar system for chlorophyll-a measurement in coastal waters. LED modules were developed for widefield microscopy [48], fluorescence spectroscopy [49,50,51,52], and atmospheric applications [53,54]. To date, there are no reported studies on the use of an LED transmitting module for an algal fluorescence monitoring system. Furthermore, this study seeks to correlate the measured range-resolved fluorescence lidar peak intensity with existing methods such as absorbance (optical density) using absorbance spectroscopy, excitation–emission matrix (EEM) chlorophyll-a pigment using fluorescence spectroscopy, and corrected chlorophyll-a measurements.

In this study, information on the use of a portable LED fluorescence lidar system for preliminary algal detection in water quality monitoring is provided. Specifically, the conducted preliminary algal analyses using a pulsed LED fluorescence lidar system for chlorophyll measurements was employed using varying Spirulina concentrations. The LED fluorescence lidar system can detect fluorescence emission of chlorophyll at the 385 nm excitation wavelength. Chlorophyll is found in all algal species, and the minimum detection of the developed LED fluorescence lidar system is 20 mg/L based on the experimental results conducted. With the initial results presented in this paper, the researchers aim to develop a lidar system that can differentiate the occurrence and growth phases of organisms with additional information on the organic matter contribution from other interactions. This study using the developed LED fluorescence lidar system can be helpful in water planning and management by both the public and private sector.

## 2. Theory

All optical methods of algae detection employ the spectral properties of the primary light-absorbing pigments, which are present in different algal species in different proportions. The design of the pulsed LED fluorescence lidar system were based on the following concepts. These equations were used as basis for the chlorophyll-a measurement needed in algal growth monitoring.

### 2.1. Absorbance

The fraction of a parallel beam of light absorbed by a sample is independent of the intensity of the incident beam and it is related to the concentration of the absorbing system as given by the Beer–Lambert Law [55]. The equation shows that

(1) $$\log_{10}\frac{I_{o}}{I}=Ecl$$

where I is the intensity of transmitted light (counts), $I_{o}$ is the intensity of incident light (counts), E is the molecular extinction coefficient (M^−1^ cm^−1^), c is the concentration in (M), and l is the pathlength of the sample (cm). The quantity $\log_{10}\frac{I_{o}}{I}$ is known as the absorbance (or optical density) of the sample.

### 2.2. Fluorescent Form of the Lidar System

For algal mapping, the algae and scattering material are assumed to be uniform in depth in the water. In this study, the correction factors for surface reflectivity and atmospheric attenuation for ranges less than 1 km are negligible [56]. The range-resolved fluorescence lidar signal is directly proportional to the chlorophyll-a concentration of algae as shown by

(2) $$\log\left(E\left(\lambda_{F},\;R\right)R^{2}\right)=g\left(\lambda_{F},\;\lambda \right)C_{algae}$$

where $E\left(\lambda_{F},\;R\right)R^{2}$ is the range-resolved fluorescence lidar signal (counts·m^2^), $g\left(\lambda_{F},\;\lambda \right)$ is the correlation coefficient, and $C_{algae}$ is the concentration of the algae (molecule/m^3^) observed. The correlation coefficient is composed of the receiver efficiency, receiver area, and receiver wavelength bandwidth. Cross sections of the fluorescence at 680 nm per chlorophyll-a molecule (*λ_F_*) were obtained in the algae when excited by LED excitation wavelength (*λ*), the water attenuation coefficients at LED excitation wavelength (385 nm) and at the fluorescence emission wavelength (680 nm), and the refractive index of water. These coefficients were assumed constant in the duration of the experiment since there are no changes in the composition of the transmitting and receiving systems. The weight of the algal biomass of Spirulina is the only variable that was changed during the duration of the fluorescence signal collection. With these assumptions, a linear relationship will be discussed further between the range-resolved fluorescence lidar signal and the concentration of the chlorophyll-a in algae.

## 3. Materials and Methods

**Figure 1:**
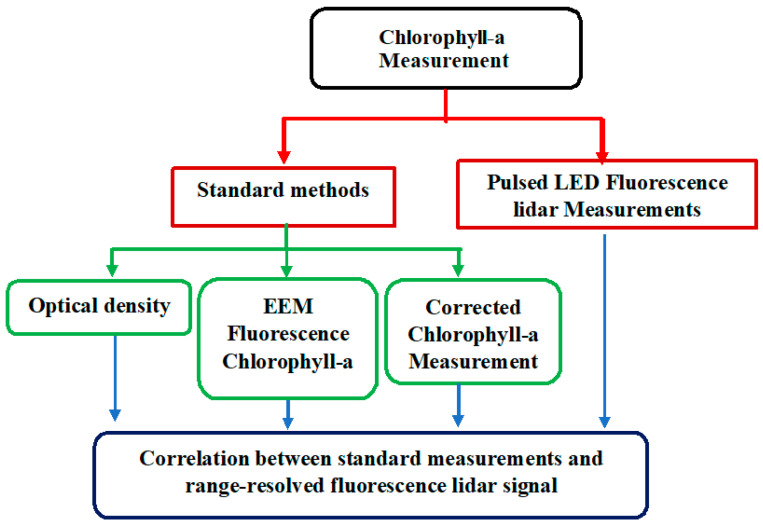
Chlorophyll-a measurements conducted using Spirulina concentration. The standard methods are optical density, EEM fluorescence chlorophyll-a analysis, and corrected chlorophyll-a measurements. These methods were correlated with the range-resolved fluorescence lidar signal. All measurements were observed at 680 nm for chlorophyll-a and 700 nm for turbidity measurements.

In this section, the algal preparation and data collection process will be described. To identify and to correlate the range-resolved fluorescence lidar signal, the different standard methods of chlorophyll-a measurement in algal growth monitoring used are: (i) optical density, (ii) EEM chlorophyll-a analysis, and (iii) corrected chlorophyll-a measurement. Figure 1 shows the schematic flow of the methods performed in this study.

### 3.1. Spirulina Concentration

Spirulina have chemical compositions containing proteins; carbohydrates; essential fatty acids; vitamins; minerals; and pigments such as carotenes, chlorophyll, and phycocyanin [57]. A commercialized AZTEC Spirulina powder (AZTEC Food growers Corp., Rizal, Philippines) was weighed with the following freeze-dried algal biomass: 10 mg, 50 mg, 125 mg, 250 mg, 375 mg, 500 mg, 625 mg, 750 mg, 875 mg, and 1000 mg. All freeze-dried samples were mixed with 500 mL of deionized water. Sample preparations were conducted in a dark room (10% light; 25 °C room temperature) to limit exposure to visible light [58].

### 3.2. Development of Portable LED Fluorescence Lidar System

#### 3.2.1. Aquarium Tank System

**Figure 2:**
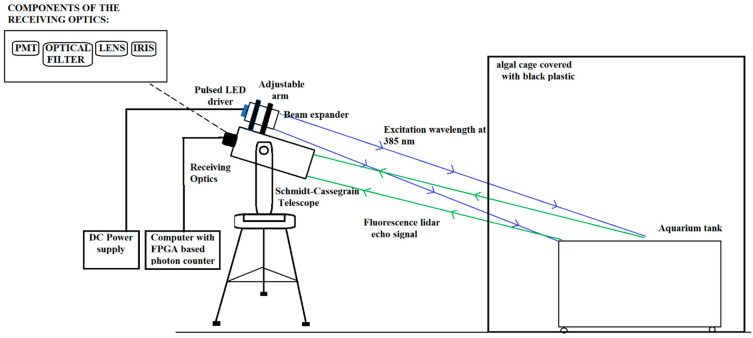
Schematic diagram of the pulsed LED fluorescence lidar system.

The design and construction of the algal aquarium tank system was carried out in De La Salle University Manila (14°33′58.6″ N, 120°59′32.0″ E). An aquarium tank was fabricated out of glass for the algal samples (Figure 2). It has dimensions of 0.5 m (L) × 0.5 m (W) × 0.5 m (H) and a glass thickness of 6 mm. It is divided into four equal partitions with 0.25 m (L) × 0.25 m (W) × 0.5 m (H) each. For each algal concentration, three out of four compartments were used for the three algal replicates. The concentration of the algae in each of the three replicates increases based on the algal biomass. The remaining compartment was used for blank sample measurements. The blank sample used in this study is deionized water. Blank samples are the most common solvent in the samples [59]. The tank is designed to be moved easily as the replicates and blank samples are being measured using the fluorescence lidar system.

#### 3.2.2. Receiving and Transmitting Systems

The EEM output with the different observed peaks suggests much information on the potential of a multi-spectral detection of water quality in the fluorescence spectrum. However, the designed lidar only focused on the detection of chlorophyll-a fluorescence at emission wavelength of 680 nm and turbidity measurements at 700 nm. The detection peak was based on the pigment EEM of Spirulina concentrations.

The schematic diagram shows the transmitting and receiving components of the pulsed LED fluorescence lidar system (Figure 2). The tabletop telescope has a height of 40 cm, the length of the telescope is 35 cm with a diameter of 12 cm. The distance between the telescope and the holder is 2.5 cm. The diameter of the LED circuit holder is 7 cm. It consists of a pulsed LED module with a 385 nm (Nichia, NCSU034C, Anan, Japan) UV LED and a trigger output. A Schmidt–Cassegrain telescope (Kenko Sky Explorer SE-AT90mm) was used for light collection and detection using bandpass filters at 680 ± 10 nm (Thorlabs, FB680-10, Newton, NJ, USA) for chlorophyll-a concentration and 700 ± 10 nm (Thorlabs, FB700-10, Newton, NJ, USA) for turbidity measurements, and a photomultiplier tube (Hamamatsu Photonics, R6358, Hamamatsu, Japan). A photomultiplier tube is useful for light detection of very weak signals and amplifies the electrons generated by a photocathode exposed to a photon flux. An FPGA-based high-speed photon counting board (Trimatiz Co., Ltd., Photon Tracker, Chiba, Japan) was utilized. The board has a four-channel input which is convenient for multiple channel detection, and it can be synchronized with a pulsed beam oscillation by a trigger-in port. The system lock is 500 MHz with power consumption of 7 W, which is approximately 2 W for each channel. The highest resolution defined by the BIN width is 5 ns which is equal to a range resolution of 0.75 m. The minimum summation time for data transfer to a computer is up to 0.2 s [54]. The portable LED fluorescence lidar system was positioned 5 m away from the algal tank. This only allows the detection of the Spirulina concentration in each tank. The three tanks are also covered in a matte black board to remove possible detection from other tanks. A summary of the transmitting and receiving components of the LED fluorescence lidar system is listed in Table 1.

The developed pulsed LED fluorescence lidar system is compact and portable which is easier to transport for field measurements. A separate power supply was used for the pulsed LED module and the photomultiplier tube wherein portable generators can also be used as a source. The lidar system uses biaxial optics for the transmitter and the receiver. From the specifications of the portable LED fluorescence lidar system listed in Table 1, the optical efficiency between the transmitting and receiving energies is less than 30%.

### 3.3. Fluorescence Spectroscopy Measurements

**Figure 3:**
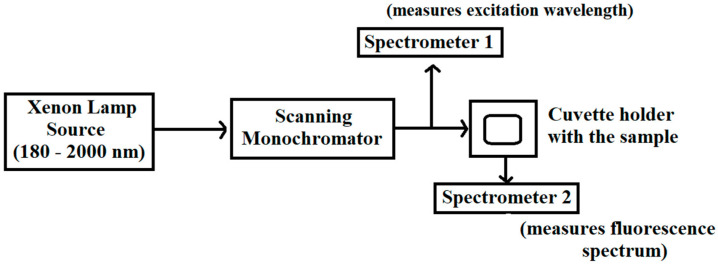
Ocean Optics fluorescence spectroscopy set-up at EARTH lab. The arrow represents the direction of the light.

The investigation of fluorescence characteristics of algal organic matter with the use of UV–vis excitation spectrum is useful in fluorescence lidar monitoring. In this study, excitation–emission fluorescence set-up was assembled in the EARTH Lab, De La Salle University Manila. An Ocean Optics spectrometer and Xenon lamp were operated. Figure 3 shows the schematic diagram of the fluorescence spectroscopy. The experimental set-up was first used in the water quality characterization and fluorescence measurements [12,13].

Using a micropipette, one (1) mL of Spirulina samples at varying concentrations was transferred into 10 mm UV–vis cuvettes and were stored at room temperature. A 105 W high-power Xenon lamp source (Ocean Optics HPX-2000, New York, NY, USA) ranging from 180 to 2000 nm was connected and controlled using a scanning monochromator (Ocean Optics MonoScan 2000, New York, USA). The scanning monochromator varies the excitation wavelength of the lamp source with a 5 nm interval. The scanning monochromator has a limiting spectral control of 250 to 800 nm with minimum interval of 1 nm. A specific excitation wavelength is provided by the monochromator as it enters the sample. Spectrometer 1 (Ocean Optics USB-4000, New York, USA) was placed after the scanning monochromator to determine the scanning spectrum of the excitation wavelength coming from the Xenon lamp. The data recorded were used for noise and data correction. The optical probe was positioned at 90° to obtain maximum fluorescence spectrum. Spectrometer 2 (Ocean Optics USB2000+ XR1-ES, New York, USA) with pre-configured UV–NIR measurements receives the fluorescence spectrum at the excitation wavelength.

### 3.4. Excitation–Emission Matrix (EEM) Data Collection and Processing

From the calibrations integrated in the spectroscopy set-up, the emission spectra of each Spirulina concentration were collected. The emission spectra at every excitation wavelength are the uncorrected fluorescence spectra. Uncorrected fluorescence EEMs from the different Spirulina samples were obtained. In this study, the excitation wavelength ranges from 250 to 450 nm. The uncorrected fluorescence spectrum ranges from 200 to 2000 nm. However, the emission (fluorescence) spectrum studied ranges from 350 to 600 nm for algal organic matter, and 600 to 800 nm for algal pigment [60,61,62]. For the data acquisition, the signal from the spectrometer was analyzed using Oceanview application. The calibration offset is 10,000 and saturation level of 5000. This calibration mode maximizes the excitation signal coming from the Xenon lamp. The scans to average per sample was set to 15 with a boxcar width of 3 as recommended in the spectroscopy manual. The integration time is approximately 20,000 ms (~0.33 min.). The Oceanview application provided a graph with emission spectrum (200 to 1025 nm) on the *x*-axis and relative intensity (photon counts) on the *y*-axis.

Fifty (50) deionized water samples served as blank samples. Spectral measurements of the blank samples were repeatedly recorded and averaged. The raw EEM data were corrected using the blank subtraction from the collected data. Rayleigh and Raman scatter lines were removed during analysis. The resulting data are the corrected fluorescence emission of the Spirulina samples at varying biomass.

Parallel factor analysis (PARAFAC) method, a three-way method that decomposes the fluorescence signature of organic matter into individual components and provides estimates of the relative contribution of each component to the total organic matter fluorescence [14,15], was employed to determine the organic and pigment components of Spirulina samples. The benefit of PARAFAC is that a more complete analysis of EEMs is possible than for traditional peak-peaking methods and additional information which may be obtained from the EEMs [16]. Moreover, PARAFAC can take overlapping fluorescence spectra and decompose them into broadly defined fluorescence components [15]. Using the EEMs of the algal organic matter (AOM), several fluorescence indices and aquatic fluorescence peaks were measured to determine the contribution of algal organic matter and chlorophyll-a pigment in water quality. Furthermore, aquatic fluorescence peaks provide protein-like and humic-like peaks at varying concentrations of Spirulina samples.

### 3.5. Absorbance Measurements

One (1) mL of each varying concentration was measured for optical density using Genesis 10uv thermospectronic set-up located at the Molecular Science Unit Laboratory, De La Salle University Manila. The optical density of Spirulina concentrations was measured at 680 nm.

Freeze-dried samples of varying algal biomasses were prepared in an aluminum foil-covered screw cap test tube. Two (2) mL of 95% ethanol was mixed in each sample and boiled at 78 °C for five minutes. After boiling, the sample rest for 24 h in a dark room at 4 °C. After 24 h, the sample was mixed thoroughly and was transferred in a centrifuge tube. The sample was centrifuged for 10 min at 5000 rpm. After the process, the supernatant was collected. Note that the experiment must be kept in darkness to minimize any pigment changes. The supernatant was prepared in a cuvette with the 95% ethanol as blank sample. The absorbance was measured at 750 nm, 663 nm, 645 nm, and 630 nm wavelengths. Corrected chlorophyll-a $\left(\frac{\mu g}{L}\right)$ measurements were calculated using the Equation (1) below [63].

(3) $$\mathrm{Corrected}\;\mathrm{chlorophyll-a}\;=\frac{[26.73\;\left(665_{a}-663_{b})\right]EF}{VL}\;$$

where 665*_a_* is the turbidity corrected absorbance at 665 nm and 663*_b_* is the turbidity corrected absorbance at 663 nm after acidification, *F* is the dilution factor, *E* is the volume of solvent used for the extraction (mL), *V* is the volume of the filtered sample (mL), and *L* is the path length (cm). Unsaturated samples are given with a dilution factor of 1. On the other hand, dilution factor of saturated sample is dependent on the number of times the sample is diluted; hence, the volume of solvent used for the extraction changes as well.

## 4. Results

This section discusses the preliminary measurements of chlorophyll-a pigment using excitation–emission matrices. It also describes the different methods used in measuring chlorophyll-a pigment such as optical density, corrected chlorophyll-a, EEM chlorophyll-a pigment, and the range-resolved fluorescence lidar signal at 680 nm. Correlation between these measurements is discussed in the last portion of this section.

### 4.1. Chlorophyll-a Pigment EEMs

**Figure 4:**
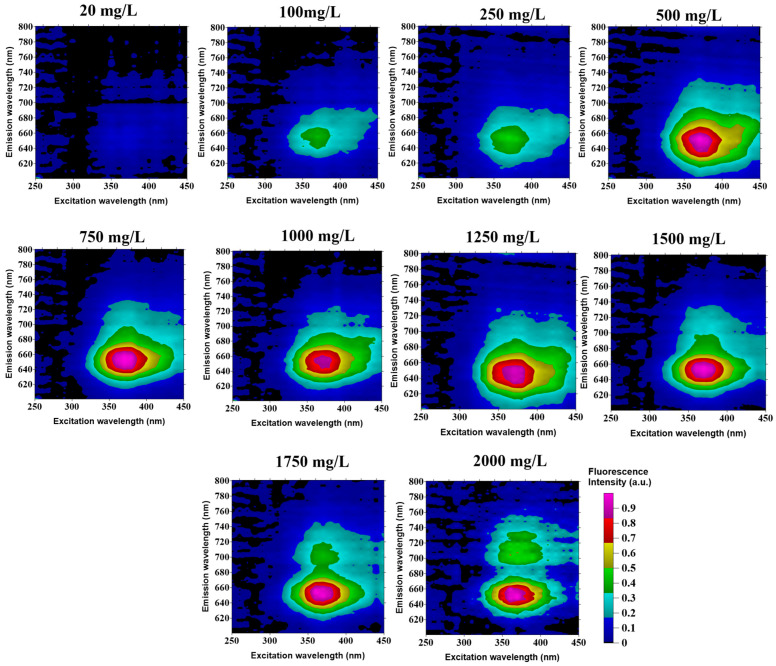
Algal pigment EEMs of commercialized AZTEC Spirulina at varying concentrations.

A single absorption spectrum of Spirulina under white light showed different emission peaks [45,64]. Spirulina samples also showed different fluorescence pigment EEMs, such as phycocyanin and chlorophyll spectrum at varying freeze-dried algal biomass. Figure 4 shows the fluorescence pigment EEMs of Spirulina at varying concentrations. The fluorescence pigment EEMs showed significant peaks at an excitation wavelength range of 300 to 420 nm with an emission wavelength range of 600 to 740 nm for concentrations from 20 to 250 mg/L. The central peak on this region is observed at 650 nm, which is known as the phycocyanin pigment [65,66]. However, the presence of another peak is observed from 1750 to 2000 mg/L Spirulina concentration.

**Figure 5:**
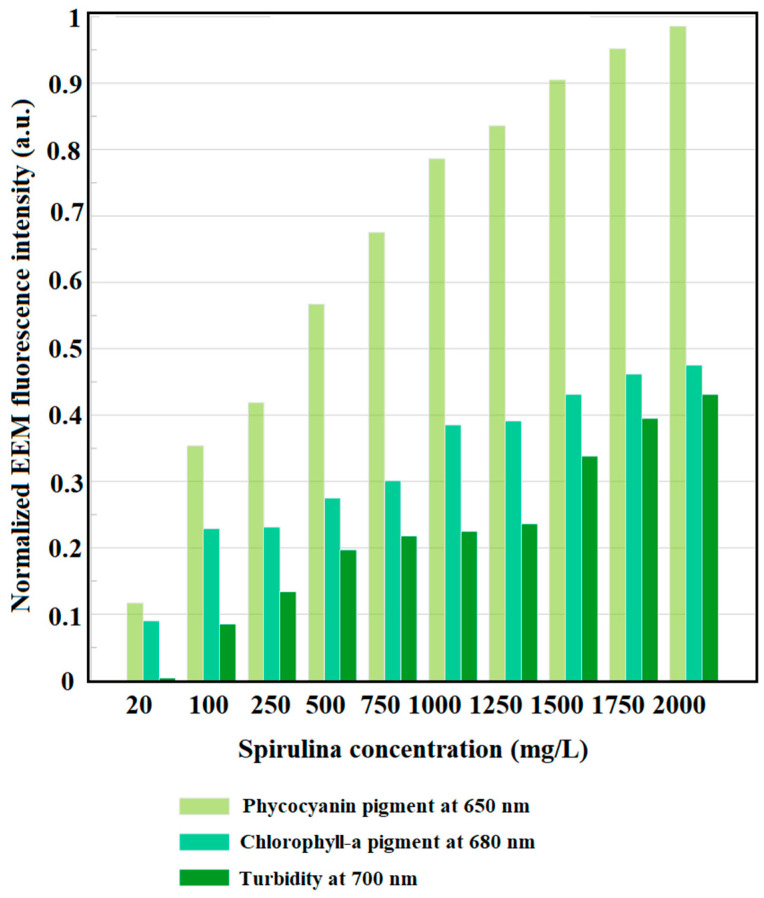
Normalized values of the pigments at varying commercialized AZTEC Spirulina concentration.

There are two pigments that were extracted from the fluorescence pigment EEMs of Spirulina—such as phycocyanin pigment at emission wavelength of 650 nm and chlorophyll-a pigment at emission wavelength of 680 nm (Figure 5). As observed high phycocyanin pigment values are measured in the different freeze-dried algal biomass samples. The difference in the phycocyanin pigment is relatively high compared to chlorophyll pigment since all blue-green alga have higher phycocyanin contents compared to other pigments [67].

### 4.2. Range-Resolved Fluorescence Lidar Signal Measurement

**Figure 6:**
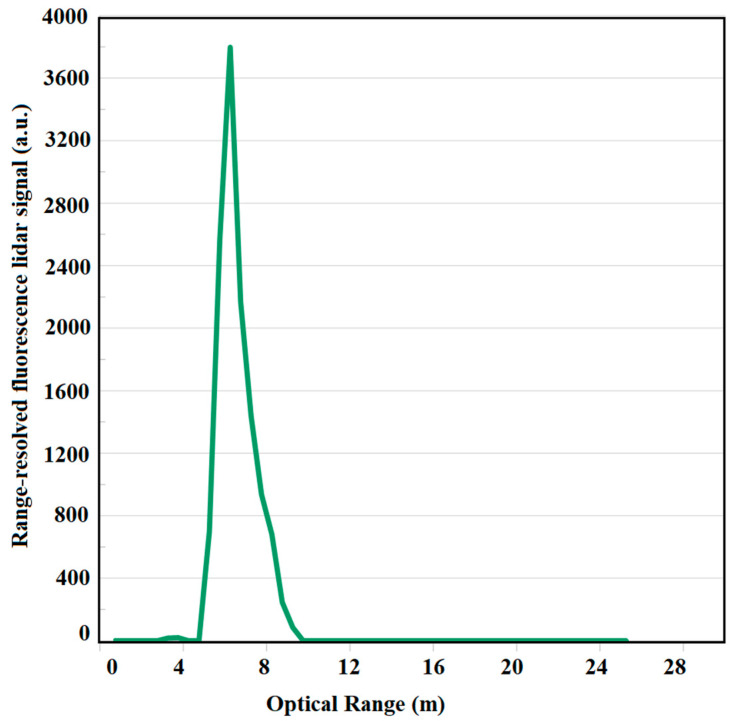
Range-resolved profile at 20 mg/L Spirulina concentration using the pulsed LED fluorescence lidar system at an excitation wavelength of 385 nm and an emission wavelength at 680 nm.

Based on the optical density and EEM measurements, the chlorophyll-a monitoring of Spirulina is conducted using 680 nm fluorescence detection. Different concentrations of Spirulina at 5, 10, 15, and 20 mg/L were measured using the LED fluorescence lidar system. Using one-way ANOVA, it was found out that there are no significant changes among the said concentrations. Hence, the minimum concentration that the LED fluorescence lidar system can detect is 20 mg/L. Figure 6 shows a sample of the range-resolved profile of Spirulina at 20 mg/L concentration. The intensity of the range-resolved fluorescence lidar signal is 3007.81 a.u. The range-resolved profile shows the whole columnar depth of the tank which represents the fluorescence volume of the Spirulina at 20 mg/L concentration.

**Figure 7:**
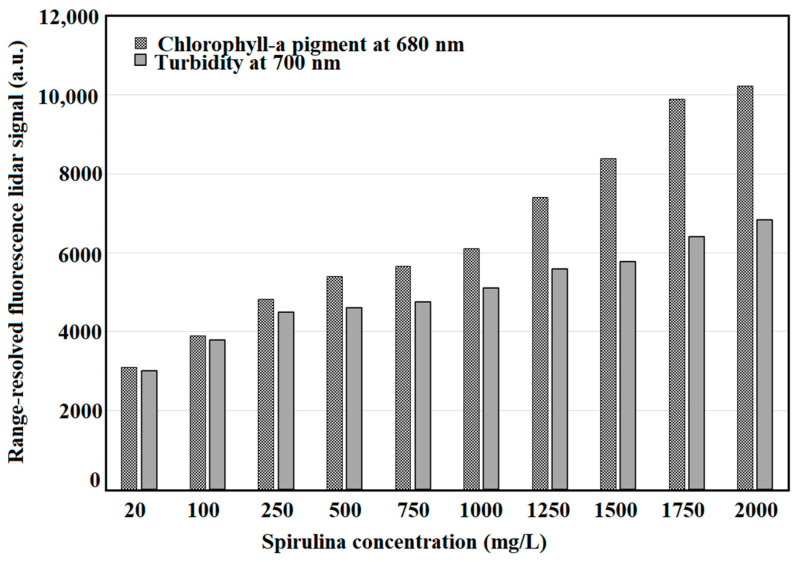
Fluorescence lidar intensity peaks of chlorophyll-a (680 nm) and turbidity (700 nm).

The lidar profile of 20 mg/L is the same for other concentrations used in this study. Figure 6 is the typical fluorescence lidar profile for these measurements. Turbidity measurements were also conducted at 700 nm fluorescence detection. Figure 7 shows the different fluorescence peaks for chlorophyll-a and turbidity measurements at 680 nm and 700 nm, respectively. The optical range for 680 nm and 700 nm measurements is the same.

The peak intensity value of the range-resolved fluorescence lidar profile was used to correlate with the EEM chlorophyll-a concentration, optical density at 680 nm, and the corrected chlorophyll-a concentration. The minimum range-resolved fluorescence lidar signal is observed at 20 mg/L Spirulina concentration with intensity of 3007.81 a.u. On the other hand, the highest range-resolved fluorescence lidar signal is observed at 2000 mg/L Spirulina concentration with intensity of 6835.92 a.u. One-way ANOVA testing was conducted to determine the variance of each Spirulina replicate. The F-value was measured at 0.004, which is found to be within the 95% level of significance. The observed effect size is small which indicates that the magnitude between the replicates is small. It means that the group explains 0.003% of the variance from the means, which is like R^2^, a linear regression. Using Tukey HSD/Tukey Kramer test [68,69], the means of any replications of Spirulina concentration showed no significant difference.

### 4.3. Correlations between Measurements

Dry weight measurements are commonly used in estimating algal biomass for different microalgae. Absorbance or optical density measurements of varying freeze-dried algal biomass of Spirulina at 680 nm emission wavelength was conducted. The absorbance ranges from 0.002 to 0.495 from 20 mg/L to 2000 mg/L of Spirulina concentration. The R^2^ value is 0.97, which shows direct correlation between the spirulina concentration and the optical density. The corrected chlorophyll-a is achieved with turbidity correction after acidification and concentration analysis. This represents the estimated concentration of chlorophyll-a in each freeze-dried Spirulina biomass. The corrected chlorophyll-a measurement showed a direct polynomial relationship with the commercialized AZTEC Spirulina concentrations. Varying dilution factors were used before acidification since the limit of the absorption spectrometer is 3.

**Figure 8:**
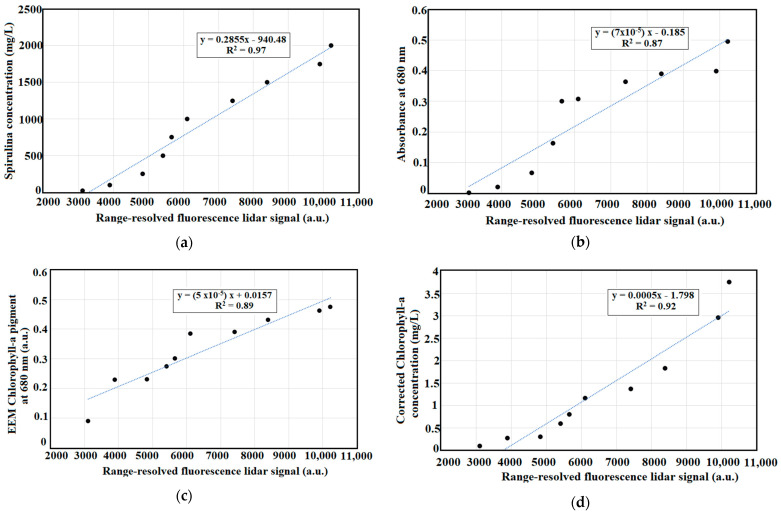
Scatter plots of range-resolved fluorescence lidar peak intensity and: (**a**) Spirulina concentration, (**b**) absorbance at 680 nm, (**c**) EEM chlorophyll-a pigment, and (**d**) corrected chlorophyll-a concentration.

Figure 8 shows the summarized chlorophyll-a measurements between range-resolved fluorescence lidar peak intensity and: (a) Spirulina concentration, (b) absorbance at 680 nm, (c) EEM chlorophyll-a pigment, and (d) corrected chlorophyll-a concentration. The absorbance measurements at 680 nm varies from 0.002 to 0.495. The chlorophyll-a pigment extracted from EEM pigment analysis ranges from 0.004 to 0.431. Likewise, the range-resolved fluorescence lidar signal of Spirulina concentration increases starting from 3007.813 to 6835.920 a.u. Range resolved fluorescence measurements of Spirulina concentration showed high correlations with Spirulina concentration (R^2^ = 0.97), absorbance at 680 nm (R^2^ = 0.87), EEM chlorophyll-a pigment (R^2^ = 0.89), and corrected chlorophyll-a concentration (R^2^ = 0.92). The study is a good reference in the development of a portable fluorescence lidar system in measuring chlorophyll-a for algal growth monitoring. The lidar system was only tested using varying concentrations of Spirulina powder at night conditions; however, it pioneers the development of a lidar system with a unique transmitting system. This pulsed LED fluorescence lidar system provides a new technique of estimating chlorophyll-a concentration of algae in natural waters.

## 5. Discussion

This section discusses the importance of the study in the development of a fluorescence lidar system for chlorophyll-a measurement. It deals with the importance and relevance of this study in algal growth and water quality monitoring in natural waters.

### 5.1. Pulsed LED Fluorescence Lidar System

The EEMs were very useful in the design of the LED fluorescence lidar system (Figure 4). It was used to identify pairs of excitation and detection (fluorescence) wavelengths that could be used in the fluorescence lidar experiment. There are important features of the pulsed LED fluorescence lidar system that contributed to the development of this algal monitoring device. The biaxial optical system of the fluorescence lidar does not require precise alignment [54]. The holder of the transmitter was also designed and printed using an Ender-3 3D printer with acrylonitrile butadiene styrene (ABS) filament cable. ABS filament is an amorphous polymer with no crystallite. It is one of the most used materials in the fused filament fabrication with its relatively low glass transition temperature and excellent processing properties [70]. The adjustable holder was designed with fine and coarse adjustments which enhances the overlap from the pulsed LED source and the telescope.

The LED light source used high repetition frequency and low pulse power. This requires that the output power needs to be higher than 100 mW. By taking into consideration the optical properties of the LED, a lamp-type LED at 385 nm was selected to ensure its portability of the transmitting system. In general, an LED has the same quick response as a laser diode (LD); however, LEDs usually provide more choices for illuminance and wavelength specifications. Due to its low pulse power ability, the LED module is eye-friendly compared to laser diodes and compact lasers. This allows the operator of the LED fluorescence to adjust the transmitting system of the set-up without possible damage to their skin or eyes. The pulsed LED fluorescence lidar system was only tested during nighttime conditions to limit factors affecting the results of the study such as background noise and light coming from the sun. In the future, the researchers plan to address this concern to further widen the capability of the developed pulsed LED fluorescence lidar system.

### 5.2. Chlorophyll-a Measurements

Since optical density measurements and dry weights measurements proved to be a convenient indirect measurement of biomass concentration in microalgae analysis [71,72,73], the prepared Spirulina concentration ranging from 20 to 2000 mg/L showed high linear correlation (R^2^ = 0.97) with range-resolved fluorescence lidar peak intensity. With the increasing concentration of Spirulina, the range-resolved fluorescence lidar signal was adapted with small differences in magnitudes between replicates. According to Barbini et al. (1998), the chlorophyll pigment showed the deconvoluted peak at 680 nm with respect to the LIF intensity [41]. This is highly observed in the EEM of 1750 and 2000 mg/L concentrations of Spirulina (refer to Figure 4). Fluorometric data can provide a wide range of spectral values that can be used to estimate different pigments concentrations in natural waters [44,74]. The use of EEM fluorescence measurements in estimating chlorophyll-a concentration showed the highest correlation with R^2^ = 0.97.

To further validate the range-resolved fluorescence lidar signal data, corrected chlorophyll-a concentration (refer to Equation (3)) was calculated to show extracted chlorophyll-a pigment from Spirulina concentration [72,73]. From the variance in optical densities, the estimated chlorophyll-a concentration of Spirulina ranges from 0.10 to 3.75 mg/L. This shows positive correlation coefficient of R^2^ = 0.92 between the range-resolved fluorescence lidar peak intensity at 680 nm and the corrected chlorophyll-a concentration [75,76]. Turbidity analysis was integrated in the chlorophyll-a measurement to incorporate the cloudiness or decrease in transparency in water. Low turbidity means less light will be scattered from its original direction. These means that particles—such as silt, clay, algae, and organic matter—may enable the detection of these particles in water [77,78,79]. Using the developed fluorescence lidar system, turbidity measurements were conducted at 700 nm [80,81,82]. Additionally, the fluorescence lidar peak near 700 nm in the fluorescence spectrum correlated strongly with the chlorophyll-a concentration [83,84,85]. This is the first study that compared range resolved fluorescence peaks of Spirulina using a new developed lidar system with absorbance, fluorescence, and corrected chlorophyll-a measurements.

**Figure 9:**
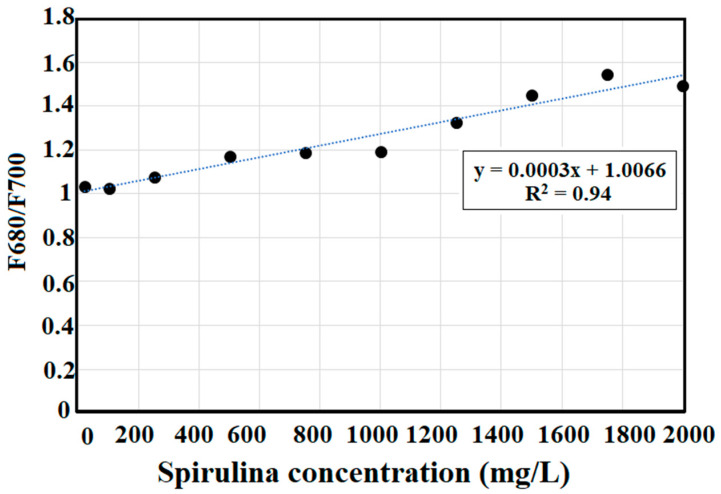
Scatter plot of Spirulina concentration and F680/F700 lidar ratio.

Chlorophyll fluorescence parameters is the extracted lidar fluorescence intensity ratio at 680 nm to that of 700 nm (F680/F700) [86]. This ratio is compared to the absolute concentration of chlorophyll-a and optical density measurements and EEM fluorescence peaks. Turbidity analysis was integrated in the chlorophyll-a measurement to incorporate the cloudiness or decrease in transparency in water. Low turbidity means less light will be scattered from its original direction. Figure 9 shows the scatter plot measurements between the lidar fluorescence ratio (F680/F700) and the Spirulina concentration.

**Figure 10:**
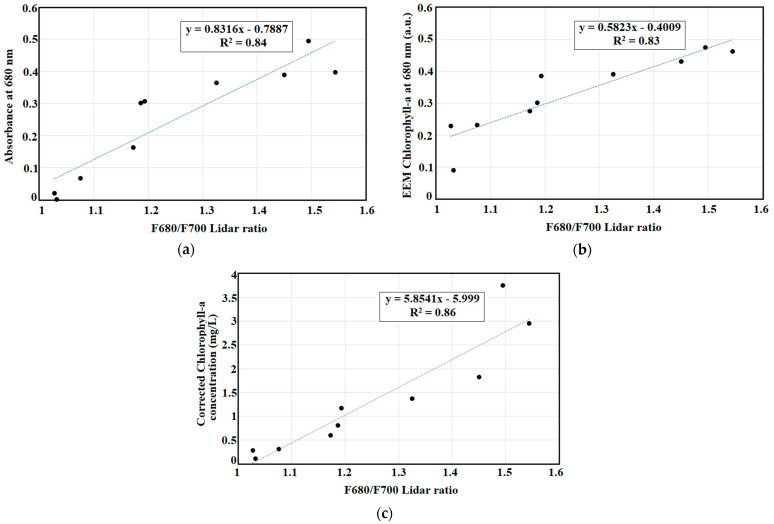
Scatter plots of F680/F700 lidar ratio and: (**a**) absorbance at 680 nm, (**b**) EEM chlorophyll-a pigment at 680 nm, and (**c**) corrected chlorophyll-a concentration.

F680/F700 provides a measurement of chlorophyll-a concentration by incorporating turbidity measurements in understanding algal growth monitoring. This lidar ratio was correlated with absorbance at 680 nm (R^2^ = 0.84), EEM chlorophyll pigment at 680 nm (R^2^ = 0.83), and corrected chlorophyll-a pigment (R^2^ = 0.86) as shown in Figure 10.

The positive correlation between the lidar-derived and laboratory-derived chlorophyll-a measurements suggest good potential of the developed LED fluorescence lidar system in algal growth monitoring. With the successful measurement of chlorophyll-a and turbidity using Spirulina concentration, we opted to improve the calibration of the lidar system and its seasonal application to natural waters.

## 6. Conclusions

A fluorescence LED lidar system was developed for chlorophyll-a measurement. It was constructed using a biaxial optics design for the transmitting and receiving systems. For the transmitting system, the pulsed 385 nm LED circuit has a bandwidth of 10 ns with a repetition frequency of 475 kHz. The maximum power observed of the pulsed LED is 830 mW. For the receiving system, 680 and 700 nm bandpass filters were used to measure the fluorescence backscatter signal of Spirulina concentration for the chlorophyll-a and turbidity, respectively. The beam divergence of the transmitter is 5 mrad. The acquisition device used in this study is a photomultiplier tube with a high photon counting device with a resolution of 0.75 m.

A linear regression trend was observed between the maximum value of range-resolved fluorescence lidar signal and increasing concentrations (R^2^ = 0.97) of Spirulina. This is supported with the corrected concentration of chlorophyll-a (R^2^ = 0.92), the EEM pigment measurement of chlorophyll-a at 680 nm (R^2^ = 0.89), and optical density (R^2^ = 0.87) of Spirulina concentration when compared with the range-resolved fluorescence lidar signal. Using turbidity measurements at 700 nm, the F680/F700 lidar was computed. It showed good correlation with Spirulina concentration (R^2^ = 0.94), absorbance at 680 nm (R^2^ = 0.84), EEM chlorophyll-a at 680 nm (R^2^ = 0.83), and corrected chlorophyll-a concentration (R^2^ = 0.86).

Important features of the LED fluorescence lidar system include the optics design, as well as a compactness which helps researchers to measure chlorophyll-a pigment in algal growth monitoring with limited optical alignment and eye-friendly pulse power. The developed LED fluorescence lidar system was found to be useful in estimating the chlorophyll-a concentration of Spirulina. The result from this study is unique with its transmitting and receiving properties of the pulsed LED fluorescence lidar system. To further understand its full capacity, it is suggested that the portable lidar system must be tested on algae in natural waters.

## Figures and Tables

**Table 1:** Specifications of the LED fluorescence lidar system.

Transmitter	
LED Name/Brand	Nichia, NCSU034C
Wavelength	385 nm
Peak power	830 mW
Resolution	1.2 m
Bandwidth	10.92 ns
Repetition	500 kHz
Beam diameter	50 mm*Φ*
Beam divergence	5 mrad
Receiver	
Telescope	Schmidt–Cassegrain
Beam diameter	100 mm*Φ*
Beam divergence	3 mrad
Bandpass filters:	
At 680 nm:	
-Thorlabs (FB680-10)	680 nm ± 5 nm
At 700 nm:	
-Thorlabs (FB700-10)	700 nm ± 5 nm
Detection device	Photomultiplier tube, Hamamatsu (R3650P)
Photon Counting Board	
Photon Counting Device/Brand	Spartan 6 (FPGA device) Trimatiz Co., Ltd., Photon tracker
System lock	550 MHz
BIN Width	5 ns (0.75 m)
BIN length	50
Acquisition count	167,777,214 (max)
Trigger Input Threshold level	300 mV
